# Supplementary material for: Improving Access to Radiotherapy Services in Gauteng: Quantitative Analysis of Key Time Intervals from Diagnosis to Treatment
Source: Int J Environ Res Public Health. 2025 Apr 2;22(4):544. doi: 10.3390/ijerph22040544 (PMC12026978; doi:10.3390/ijerph22040544)
Supplement: Supplementary file 1 [file ijerph-22-00544-s001.zip › Ramashia Data Collection Sheet.pdf]

Quantitative Public Radiotherapy Record Review

**Data sample :**	Research Nr.		
**Instruction**	Mark with an X where applicable		
**SECTION A: BIOGRAPHICAL DATA**	**A1.1. Hospital type**	Public	**1**
		Private	**2**
	**A1.2. Hospital name**	CMJAH	**1**
		SBAH	**2**
		BMOC	**3**
		SOC	**4**
		WDGO	**5**
		UO	**6**
	**A2. Patient gender**	Female	**1**
		Male	**2**
		Other	**3**
	**A3. Age**	18-25	**1**
		26-35	**2**
		36-45	**3**
		46-55	**4**
		56-65	**5**
		66-70	**6**
		>70	**7**
**SECTION B: CANCER DIAGNOSIS**	**B1. First hospital presentation date (seeing specialist)**	yyyy/mm/dd	
	**B2. Name of the referral hospital**		
	**B3. Date of cancer diagnosis- biopsy**	yyyy/mm/dd	
	**B4. Cancer type Primary site**	Breast	**1**
		Cervical	**2**
		Gastro-Intestinal	**3**
		Head & Neck	**4**
		Prostate	**5**
	**B5. Stage of disease**	Stage 1	**1**
		Stage 2	**2**
		Stage 3	**3**
		Stage 4	**4**
	**B6.1. Chemotherapy**	Yes	**1**
		No	**2**
	**B6.2. If yes, the date of chemotherapy**	yyyy/mm/dd	

	**B7.1. Surgery**	Yes	**1**
		No	**2**
	**B7.2. If yes, the date of surgery**	yyyy/mm/dd	
	**B8. Date of last follow up**	yyyy/mm/dd	
	**B9.1. Death**	Yes	**1**
		No	**2**
	**B9.2. If yes, the date of death**	yyyy/mm/dd	
**SECTIONC: RADIOTHERAPY**	**C1. Type of cancer treatment**	Neoadjuvant	**1**
		Adjuvant	**2**
		Concurrent	**3**
	**C2. Type of radiotherapy**	Radical	**1**
		Palliative	**2**
	**C3. Dose fractionation**	1.8 Gy	**1**
		2.0 Gy	**2**
		3.0 Gy	**3**
		Other (specify) ____________________	**4**
	**C4. Radiotherapy simulation date**	yyyy/mm/dd	
	**C5. Radiotherapy start date**	yyyy/mm/dd	
	**C6. Radiotherapy end date**	yyyy/mm/dd	
	**C7.1 Treatment plan change**	Yes	**1**
		No	**2**
	**C7.2 If yes, specify the reason for the plan change.**	Progressive disease	**1**
		Poor response	**2**
		Other (specify) ____________________	**3**
**SECTION D: DURATIONS**	**D1. The duration between first hospital presentation date and**	Less than 1 month (<1)	**1**
		1-3	**2**

	**cancer diagnosis (in months)**	4-6	**3**
		7-9	**4**
		10-12	**5**
		>12	**6**
	**D2. The duration between cancer diagnosis and radiotherapy simulation (in months)**	Less than 1 month (<1)	**1**
		1-3	**2**
		4-6	**3**
		7-9	**4**
		10-12	**5**
		>12	**6**
	**D3. The duration between radiotherapy simulation and Radiotherapy start date (in weeks)**	<1	**1**
		1	**2**
		2	**3**
		>2	**4**
